# A MECHANISM OF ACTION OF HUMAN-ORIGIN PROBIOTIC COCKTAIL IN ATTENUATING ALZHEIMER’S DISEASE PATHOLOGY

**DOI:** 10.1093/geroni/igad104.3805

**Published:** 2023-12-21

**Authors:** Santosh Kumar Prajapati, Shaohua Wang, Sidharth Mishra, Shalini Jain, Hariom Yadav

**Affiliations:** University of South Florida, Tampa, Florida, United States; Ohio University, Athens, Ohio, United States; University of South Florida, Tampa, Florida, United States; University of South Florida, Tampa, Florida, United States; University of South Florida, Tampa, Florida, United States

## Abstract

Alzheimer’s disease (AD) is a detrimental public health problem affecting millions of older adults in the USA alone, with no or limited treatment options. Emerging evidence indicates the pivotal role of gut microbiota in AD pathology, but mechanisms remain largely unknown. Also, microbiome modulators like probiotics are proven beneficial in preventing AD with little understanding of their mechanisms. Therefore, we investigated how a microbiota metabolite-short-chain free fatty acid receptor-2 (FFAR2) signaling in the intestine affects AD pathology in brain using intestinal FFAR2 knock-out APP/PS-1 mice; as well as determined if a human-origin-probiotic cocktail exhibits its actions via FFAR2 signaling. We measured changes in the microbiome, permeability in gut and blood-brain barriers (BBB), neuroinflammation, AD pathology, and cognitive behaviors. Results showed, the mice lacking FFAR2 in their intestine exacerbated AD pathology by developing abnormal changes in gut microbiome and intestinal permeability, which in turn promotes permeability in BBB and neuroinflammation. It’s also promoting microglia activation linked with increased Aβ accumulation and cognitive decline. It’s interesting to note that probiotic cocktail feeding reversed these abnormalities in AD mouse models. Further, this probiotic cocktail showed its mechanism in both FFAR2-dependent and independent manner. Overall, our findings indicate that the microbiome sensing mechanism FFAR2 signaling participates in regulating the gut-brain in AD pathology, and a human-origin probiotic cocktail improves the abnormal changes in the microbiome-intestinal & BBB permeability-neuroinflammation axis to ameliorate AD pathology. Thus, FFAR2 agonism and probiotic therapy can be viable options to prevent/ delay the risk of AD progression in older adults.

